# Supplementary material for: Exploring data literacy self-perception among Indonesian high school students
Source: PLoS One. 2025 Jan 3;20(1):e0312306. doi: 10.1371/journal.pone.0312306 (PMC11698427; doi:10.1371/journal.pone.0312306)
Supplement: Attachment — Submitted filename: 1PONE-D-24-17279_reviewer.pdf [file pone.0312306.s002.pdf]

PLOS ONE

Exploring data literacy self-perception among Indonesian high school students

--Manuscript Draft--

**Manuscript Number:**	PONE-D-24-17279
**Article Type:**	Research Article
**Full Title:**	Exploring data literacy self-perception among Indonesian high school students
**Short Title:**	Exploring data literacy among Indonesian high school students
**Corresponding Author:**	Ririn Yuniasih<br>Monash University<br>Melbourne, Victoria AUSTRALIA
**Keywords:**	data literacy; boot camps; high school; Indonesia
**Abstract:**	Data is becoming increasingly ubiquitous today, and data literacy has become an essential skill in the workplace. Therefore, it is necessary to equip high school students with data literacy skills to prepare them for further learning and employment. In the Indonesian education context, there is a growing shift towards integrating data literacy in the high school curriculum. In a pilot intervention project, academics from two leading Universities conducted data literacy boot camps for high school students from various cities in Indonesia. This paper explores students' self-perception of data literacy as a result of the skills acquired from the boot camp. Qualitative and quantitative data were collected through student surveys and a focus group discussion, and the data collected was used to analyse student perception post-intervention. We also explored what is currently lacking at schools, students' learning aspirations and learning materials they find useful for their development. The findings indicate that students are more aware of the usefulness of data literacy and its application in future studies and work after joining the boot camp. Among materials delivered at the boot camp, students benefited most from the delivery and application of basic statistical concepts and using Microsoft Excel as a tool for basic data analysis. The findings provide policy recommendations that educators and policymakers can use as guidelines for effective data literacy teaching in high schools.
**Order of Authors:**	Charanjit Kaur
	Pei Tan
	Nurjannah Nurjannah
	Ririn Yuniasih
**Additional Information:**	
**Question**	**Response**
**Financial Disclosure**<br><br>Enter a financial disclosure statement that describes the sources of funding for the work included in this submission. Review the submission guidelines for detailed requirements. View published research articles from *PLOS ONE* for specific examples.<br><br>This statement is required for submission and **will appear in the published article** if the submission is accepted. Please make sure it is accurate.	The author(s) received no specific funding for this work.

Powered by Editorial Manager® and ProduXion Manager® from Aries Systems Corporation

**Funded studies**<br>Enter a statement with the following details:<br>• Initials of the authors who received each award<br>• Grant numbers awarded to each author<br>• The full name of each funder<br>• URL of each funder website<br>• Did the sponsors or funders play any role in the study design, data collection and analysis, decision to publish, or preparation of the manuscript?<br><br>Did you receive funding for this work?	
**Competing Interests**<br><br>Use the instructions below to enter a competing interest statement for this submission. On behalf of all authors, disclose any competing interests that could be perceived to bias this work—acknowledging all financial support and any other relevant financial or non-financial competing interests.<br><br>This statement is required for submission and **will appear in the published article** if the submission is accepted. Please make sure it is accurate and that any funding sources listed in your Funding Information later in the submission form are also declared in your Financial Disclosure statement.<br><br>View published research articles from *PLOS ONE* for specific examples.	The authors have declared that no competing interests exist.

Powered by Editorial Manager® and ProduXion Manager® from Aries Systems Corporation

**NO authors have competing interests**<br><br>Enter: *The authors have declared that no competing interests exist.*<br><br>**Authors with competing interests**<br><br>Enter competing interest details beginning with this statement:<br><br>*I have read the journal's policy and the authors of this manuscript have the following competing interests: [insert competing interests here]*<br><br>* typeset	
**Ethics Statement**<br><br>Enter an ethics statement for this submission. This statement is required if the study involved:<br><br>• Human participants<br>• Human specimens or tissue<br>• Vertebrate animals or cephalopods<br>• Vertebrate embryos or tissues<br>• Field research<br><br>Write "N/A" if the submission does not require an ethics statement.<br><br>General guidance is provided below. Consult the submission guidelines for detailed instructions. **Make sure that all information entered here is included in the Methods section of the manuscript.**	The ethical aspects of this research project have been approved by Monash University Human Research Ethics Committee project ID 31980.<br>Written explanatory statement and consents of the study was informed and distributed to participant.<br>Written consents were obtained from student participants, their parents and teachers prior to data collection.

**Format for specific study types**<br><br>**Human Subject Research (involving human participants and/or tissue)**<br>• Give the name of the institutional review board or ethics committee that approved the study<br>• Include the approval number and/or a statement indicating approval of this research<br>• Indicate the form of consent obtained (written/oral) or the reason that consent was not obtained (e.g. the data were analyzed anonymously)<br><br>**Animal Research (involving vertebrate animals, embryos or tissues)**<br>• Provide the name of the Institutional Animal Care and Use Committee (IACUC) or other relevant ethics board that reviewed the study protocol, and indicate whether they approved this research or granted a formal waiver of ethical approval<br>• Include an approval number if one was obtained<br>• If the study involved *non-human primates*, add *additional details* about animal welfare and steps taken to ameliorate suffering<br>• If anesthesia, euthanasia, or any kind of animal sacrifice is part of the study, include briefly which substances and/or methods were applied<br><br>**Field Research**<br><br>Include the following details if this study involves the collection of plant, animal, or other materials from a natural setting:<br>• Field permit number<br>• Name of the institution or relevant body that granted permission	
**Data Availability**<br><br>Authors are required to make all data underlying the findings described fully available, without restriction, and from the time of publication. PLOS allows rare exceptions to address legal and ethical concerns. See the PLOS Data Policy and FAQ for detailed information.	Yes - all data are fully available without restriction

A Data Availability Statement describing where the data can be found is required at submission. Your answers to this question constitute the Data Availability Statement and **will be published in the article**, if accepted.<br><br>**Important:** Stating 'data available on request from the author' is not sufficient. If your data are only available upon request, select 'No' for the first question and explain your exceptional situation in the text box.<br><br>Do the authors confirm that all data underlying the findings described in their manuscript are fully available without restriction?	
**Describe where the data may be found in full sentences. If you are copying our sample text, replace any instances of XXX with the appropriate details.**<br><br>• If the data are **held or will be held in a public repository**, include URLs, accession numbers or DOIs. If this information will only be available after acceptance, indicate this by ticking the box below. For example: *All XXX files are available from the XXX database (accession number(s) XXX, XXX.)*.<br>• If the data are all contained **within the manuscript and/or Supporting Information files**, enter the following: *All relevant data are within the manuscript and its Supporting Information files.*<br>• If neither of these applies but you are able to provide **details of access elsewhere**, with or without limitations, please do so. For example:<br><br>*Data cannot be shared publicly because of [XXX]. Data are available from the XXX Institutional Data Access / Ethics Committee (contact via XXX) for researchers who meet the criteria for access to confidential data.*<br><br>*The data underlying the results presented in the study are available from (include the name of the third party*	All relevant data are within the manuscript and its Supporting Information files.

and contact information or URL).<br>• This text is appropriate if the data are owned by a third party and authors do not have permission to share the data.<br><br>* typeset	
Additional data availability information:	

Manuscript

Click here to access/download;Manuscript;Exploring data literacy students self-perception.docx

Exploring data literacy self-perception among Indonesian high school students

[CHARANJIT, KAUR; PEI P. TAN; NURJANNAH; RIRIN YUNIASIH]

[MONASH UNIVERSITY; MONASH UNIVERSITY; BRAWIJAYA UNIVERSITY; MONASH UNIVERSITY]

Corresponding author: CHARANJIT, KAUR

Abstract

Data is becoming increasingly ubiquitous today, and data literacy has become an essential skill in the workplace. Therefore, it is necessary to equip high school students with data literacy skills to prepare them for further learning and employment. In the Indonesian education context, there is a growing shift towards integrating data literacy in the high school curriculum. In a pilot intervention project, academics from two leading Universities conducted data literacy boot camps for high school students from various cities in Indonesia. This paper explores students' self-perception of data literacy as a result of the skills acquired from the boot camp. Qualitative and quantitative data were collected through student surveys and a focus group discussion, and the data collected was used to analyse student perception post-intervention. We also explored what is currently lacking at schools, students' learning aspirations and learning materials they find useful for their development. The findings indicate that students are more aware of the usefulness of data literacy and its application in future studies and work after joining the boot camp. Among materials delivered at the boot camp, students benefited most from the delivery and application of basic statistical concepts and using Microsoft Excel as a tool for basic data analysis. The findings provide policy recommendations that educators and policymakers can use as guidelines for effective data literacy teaching in high schools.

Keywords: data literacy, boot camps, high school, Indonesia.

Introduction

Data is becoming increasingly ubiquitous today, and data literacy is an essential skill in learning and employment. It is a fundamental skill because more organisations rely on data-driven decision-making. The Organisation for Economic Co-operation and Development (OECD, 2019) emphasises the importance of data literacy for learning in 2030 and beyond as it equips students with “the fundamental conditions and core skills, knowledge, attitudes and values that are prerequisites for further learning across the entire curriculum” (p.2). Data literacy is the ability to read, comprehend, analyse and communicate data (Erwin, 2015; Shreiner & Dykes, 2021; OECD, 2019). Students must be able to think critically while working with data and interpret the generated statistics as evidence. For students in secondary education, in particular, data literacy is crucial to pursue further education and future career (Carlson, et al., 2011; Erwin, 2015; Gebre, 2018; Schield, 1999). With the proliferation of data in the modern world, students must learn how to assess the validity and reliability of sources, as well as how to use data to draw meaningful conclusions.

Despite increasing awareness of the importance of understanding data in a broader scope, both in generating and using data, the initiatives to address this issue still need to be improved (Gebre, 2018). Current research shows that “literature and skill training on data literacy has been slow to develop” (Henderson & Corry, 2020, p.241), and there is still much space for new initiatives in this discipline area (Henderson & Corry, 2020; Kennedy-Clark et al., 2020). Most data literacy initiatives in secondary education have been focused on building students’ numeracy skills to improve their capacity to work with quantitative data (Gebre, 2018). However, the initiatives have not adequately addressed the primary emphasis of data literacy teaching in the Secondary curriculum to ensure students grasp basic statistical concepts and their real-world applications. The results of a study on data literacy in secondary science classrooms show that although students are competent in data exploration skills, they struggle with applying these skills. This is especially true when evaluating the data and creating evidence to support their arguments (Irish, Berkowitz & Harris, 2019). Similar findings were reported from a study of data literacy in Physics among Indonesian high school students (Suryadi, Mahardika, Supeno & Sudarti, 2021). According to the study, students have difficulties assessing data quality and analysing, interpreting,

implementing and evaluating data. As a result, they are not able to utilise subject-specific data to address real-world problems (Suryadi et al., 2021).

The gaps identified from existing research and literature on data literacy indicate a greater need for innovative initiatives when teaching data literacy skills in high schools. Suryadi et al. (2021) suggest integrating data literacy skills in specific subjects, such as physics, along with relevant learning materials necessary to improve students' skills in collecting, processing and analysing data. Furthermore, most of the research on data literacy research is conducted from the perspective of researchers rather than that of participants (Kennedy-Clark et al., 2020). Therefore, it is crucial that we explore the view of students when generating new insights into data literacy studies.

Within the context of education in Indonesia, the government's new Education initiative prioritises the inclusion of data literacy in the high school curriculum. According to Nadiem Makarim (2019), Minister of Education, Culture, Research and Technology, to accelerate educational innovation in Indonesia, young Indonesians must acquire data and computer literacy, including statistics, coding, and programming. The new roadmap introduced by the Minister includes an Emancipated Learning program known as *Merdeka Belajar* (Ministry of Education, Culture, Research and Technology Republic of Indonesia [MoECRT], 2021). *Merdeka Belajar* outlines the need for a curriculum based on competencies. It provides teachers and students with the autonomy to improve their teaching and learning, with a greater emphasis on technology and innovation (MoECRT, 2021).

To support the government's efforts, there needs to be greater awareness of the criticality of data literacy skills. We designed a pilot intervention project that complements *Merdeka Belajar*. The intervention comprises data literacy boot camps collaboratively organised by academics from Monash University and Universitas Brawijaya. Students in year 10 and year 11 from high schools and vocational high schools from different regions in Indonesia were invited to participate in the boot camps, aiming to accommodate students from different contexts of schools. Ninetyseven students who participated in this pilot project were located in different cities and islands of Indonesia. With this demography of participants, the boot camps were conducted virtually, allowing participation from different locations (Farr, Zeh & Abdelfattah, 2021). The participants attended two virtual sessions, two-hours long in each session, in which they learned some basic statistical concepts and their

applications in daily life. Case studies using simple data sets, such as analysing hospital quality and employees' performance, were provided to help students to apply their understanding of basic statistical concepts such as mean, median, and standard deviation.

The boot camps were designed based on the framework underlined in the Pre-K–12 Guidelines for Assessment and Instruction in Statistics Education II (GAISE II), published by the National Council of Teachers of Mathematics/NCTM (2020). According to the guidelines, statistics education should be approached as an inquiry process. In designing the boot camps, we also acknowledge that teaching introductory statistics courses in college needs a focus on statistical thinking and conceptual understanding (American Statistical Association [AMA], 2016). It is beneficial for high school students to acquire fundamental statistical concepts and applications before entering college to prepare for the transition from secondary to higher education.

This paper investigates how high school students perceive data literacy after participating in the boot camps. Qualitative and quantitative data were collected through student surveys and focus group discussions with students to capture how students perceive their ability of basic statistical concepts, data analysis, data presentation, data rationalisation and basic Excel skills. We also explored what is currently lacking at schools, students' learning aspirations and the learning materials they find valuable and helpful for their development. The findings provide policy recommendations for educators and policymakers when implementing data literacy teaching strategies.

What is the research design that authors used in this study? research and development? or mixed methods?

The framework for the study

The framework for the pilot intervention project is twofold, considering project participants are high school students transitioning into higher education. Firstly, we looked at current levels of data literacy and skills taught at participating schools. We adopted strategies proposed by Pre-K–12 Guidelines for Assessment and Instruction in Statistics Education II (GAISE II) by the National Council of Teachers of Mathematics (NCTM, 2020) in order to enhance their skills.

In the second fold, we designed learning materials for boot camps that focused on cultivating data literacy skills, statistical thinking and conceptual understanding to prepare students for tertiary education. The teaching strategies for the boot camps were drawn from the *Guidelines for Assessment and Instruction in Statistics Education (GAISE) College report 2016* (AMA, 2016).

Based on the two guidelines above, we designed the learning materials based on a problem-solving approach. This approach requires students to acquire the ability to understand and solve investigative statistical questions, collect and analyse data as well as interpret results. These components are depicted in Figure 1 below.

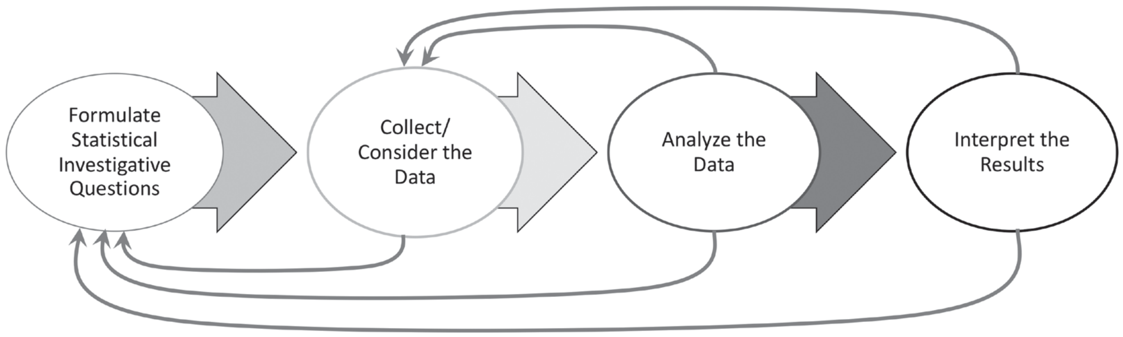

Figure 1: Statistical problem-solving process, adopted from The Pre-K–12 Guidelines for Assessment and Instruction in Statistics Education II (GAISE II), by the National Council of Teachers of Mathematics, 2020.

We also included a data challenge for participants to engage in "an investigative process of problem-solving and decision making" (AMA, 2016, p.3) and "experience with multivariable thinking" (AMA, 2016, p.3). In the data challenge, we used real-world data with a particular context and purpose (AMA, 2016) so that students would better understand basic statistical concepts learned at school.

To support student learning before and during the boot camps, we designed a Google site as a centralised repository for learning materials used during the boot camps. The site contained short videos in preparation for the boot camp activities. Research shows that videos can be an effective learning tool (Stockwell et al., 2015) as they are easily accessible, provide visual aid and help keep students engaged. In addition to videos, the Google site housed all data sets and activity sheets used during the boot camps. The framework described above sets the parameters we used to develop the instruments for data collection, including surveys and questions for focus group discussions. In the next section, we describe the measures used to collect data.

Data Collection

Further explanation needed for the instrument used in this study: how you develop the instruments, and did the authors used/adopted a conceptual approach

A Likert scale-based questionnaire was designed to measure students' perceptions towards their data literacy skills before and after the boot camps. These questions cover various aspects of students' perception and comprehension of data, practical skills and attitude towards data. The questionnaire was divided into the following categories: Basic statistical concepts, Excel skills, Data analysis skills, Perception of data literacy, Data rationalisation skills. Pre-surveys were distributed on 24 June 2022 to gather students' perception on their data literacy skills prior the boot camp. After the boot camp, post-surveys were distributed on 17 July 2022 to generate students' perception after joining the boot camp.

When measuring changes in student perceptions towards their data literacy skills, we filtered responses to include students who completed the questionnaires before and after the boot camp. Approximately forty per cent of all participants were included in our data.

In addition to the student survey, we conducted a focus group discussion with three male and two female students. This discussion aimed to explore participants' experiences and opinion about the boot camp. It allowed them to elaborate on their responses to the survey. This method is useful in generating information that can "construct descriptive and interpretive accounts of participants' experiences and meanings" (Kamberelis, Dimitriadis & Welker, 2018, p.1210). Semi-structured guiding questions were designed for the group discussion in order to explore participants' experiences and opinions about the boot camp. Five students, three males and two females, participated in the hour-long Zoom discussion. Transcripts of the discussion were then analysed to explore themes that emerge from participants' responses to the questions. The finding from this thematic analysis was then interpreted within the context of high school education in Indonesia. According to Kamberelis, Dimitriadis and Welker (2018), practice and meaning should be understood within "the contexts in which they occur" (p.1210). Furthermore, in order to understand parts of a phenomenon, we need to have a grasp of the entire phenomenon.

explain more on this, how you maintain the personal contact? was it FGD? or what? what the main issues explored?

Analysis and Results

The following statistical analysis was carried out using the R package. The Cronbach's alpha reliability test assessed the questionnaires' reliability and consistency. In addition to Cronbach's alpha, omega coefficients were used to test the consistency of the questionnaires, in which Cronbach's alpha assumption fails with unequal tau-equivalence or error variance. Because the questionnaires were on a 5-point Likert scale, the difference between the pre-post survey measures for each variable was tested using the Wilcoxon signed rank test. The magnitude of difference for the pre-post study was also examined using effect size. Furthermore, the size of the effect acts as an indicator of the workshop's effectiveness.

Results

Thirty-eight students participated in and completed pre-post Likert scale questionnaires about their perceptions of data literacy from various perspectives. Cronbach's alpha values for the pre-post survey on each data literacy component are shown in Table 1. The Cronbach’s alpha values are in the acceptable range, with a minimum of 0.94 for the post-survey and a minimum of 0.96 for the pre-survey. There is sufficient internal consistency, as indicated by the alpha and omega values, suggesting no one-dimensional questions exist.

Table 1
Descriptive statistics and reliability tests

Survey	Components	Mean	S.D.	α	ω
Pre	Basic Statistical	2.77	1.23	0.97	0.98
Post	Concept	3.33	1.15	0.97	0.98
Pre	Basic Excel Skill	3.43	1.34	0.94	0.97
Post		3.76	1.11	0.97	0.98
Pre	Data Analysis	2.70	1.28	0.94	0.97
Post		3.30	1.06	0.96	0.98
Pre	Perception of	4.14	1.09	0.96	0.98
Post	Data Literacy	3.75	1.21	0.98	0.99
Pre	Data	3.87	1.16	0.96	0.98
Post	Rationalization	3.71	1.11	0.98	0.98

Notes: S.D.: standard deviation, α: Cronbach’s alpha, ω: omega coefficient.

Figure 2 depicts the density distribution of the total score for the pre and post-survey based on students' perceptions of different categories (basic statistical concept, Excel skill, data analysis, perception of data literacy, and data rationalisation). The density distribution shows that the student's perceptions about their understanding of

basic statistical concepts and data analysis skills have significantly improved. In addition, perceptions about their basic Excel skills have improved only slightly. The only area in which there is no significant change is their perception of data literacy and their opinion of their ability to rationalise data.

Figure 2

Density distribution of the total score for the pre and post-survey based on students' perceptions of different categories

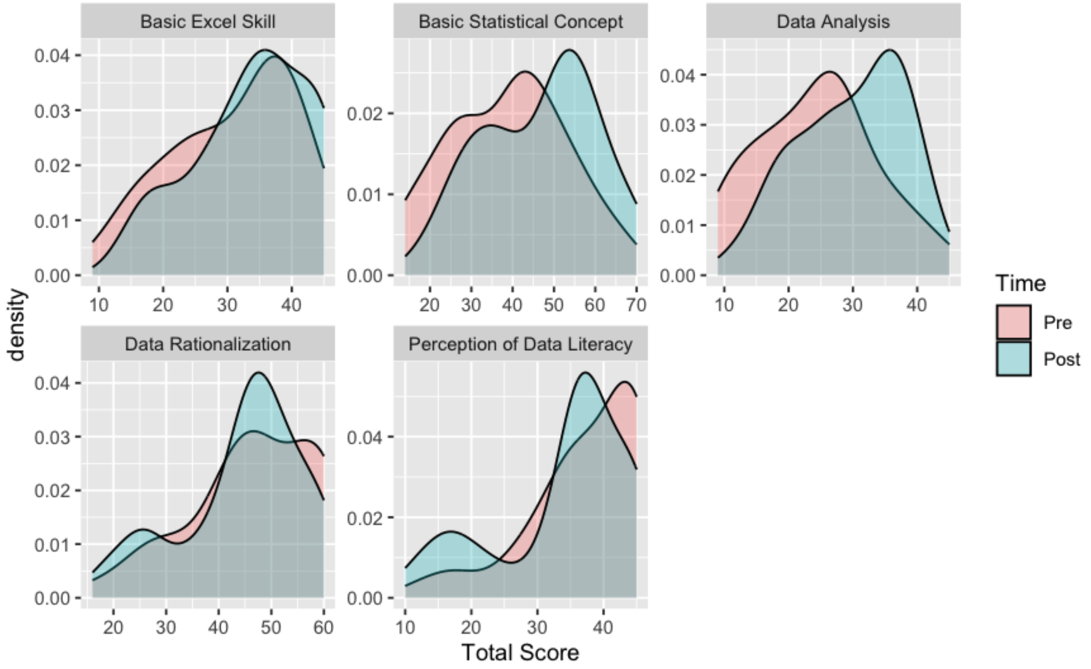

We then address the main research question, "Did students' perceptions improve after the workshop, and if so, how large is the effect?" The concept category with the most significant effect size is data analysis. Because the scores are all based on ordered Likert scales, the Wilcoxon sign rank test was used. Based on Table 2, the signed test results revealed a significant improvement from pre- to post-score on their opinion students' perception of their understanding of basic statistical concepts and data analysis (the p-values are significant at a 5 per cent level).

Table 2

Pre- and post- score of students' perceptions towards their understanding of statistical basic concepts

Components	Pre Mean (S.D.)	Post Mean (S.D.)	Effect Size	p-value
Basic Statistical Concept	2.77 (1.23)	3.33 (1.15)	0.281	0.000*
Basic Excel Skill	3.43 (1.34)	3.76 (1.11)	0.131	0.269
Data Analysis	2.70 (1.28)	3.30 (1.06)	0.293	0.010*

Perception of Data Literacy	4.14 (1.09)	3.75 (1.21)	0.160	0.343
Data Rationalization	3.87 (1.16)	3.71 (1.11)	0.084	1.000

Notes: S.D.: standard deviation; ** statistically significant at 5% level.

As a follow-up to the findings above, we analysed gender differences in perceptions on data literacy. This would help us determine potential factors that may

unclear, and some confusion with the This refers to?

contribute to one gender having a better understanding of data literacy (Rizal et al.,

I am not sure with this claim, authors need to support the argument with more references

2021). This could include education, experience, or even innate cognitive abilities. Researchers can better understand the factors contributing to differences in data analysis skills by studying gender differences in data literacy. They can also identify potential areas for improvement or intervention. Furthermore, understanding possible gender differences in data literacy can inform the development of educational and training programmes designed to help individuals improve their data analysis skills, regardless of gender. Interestingly, Table 3 shows no statistically significant difference in perceptions on data literacy between male and female students. Both genders similarly perceive the importance of different aspects of data literacy.

Table 3

Gender differences in perceptions towards understanding of statistical basic concepts

Survey	Variable	Female Mean (S.D.)	Male Mean (S.D.)	Effect Size	p-value
Pre	Basic Statistical	2.739(1.316)	2.810(1.106)	0.041	0.809
Post	Concept	3.183(1.210)	3.520(1.035)	0.186	0.251
Pre	Basic Excel Skill	3.295 (1.335)	3.605(1.325)	0.152	0.349
Post		3.609(1.139)	3.951(1.044)	0.205	0.205
Pre	Data Analysis	2.681(1.320)	2.734(1.220)	0.048	0.777
Post		3.155(1.121)	3.475(0.947)	0.162	0.320
Pre	Perception of Data	4.092(1.193)	4.191(0.956)	0.075	0.784
Post	Literacy	3.928(1.190)	3.531(1.191)	0.075	0.332
Pre	Data Rationalization	3.873(1.248)	3.856(1.044)	0.046	0.648
Post		3.699(1.122)	3.713(1.104)	0.158	0.649

It is generally believed that individuals who study science and mathematics have a more robust understanding of data literacy compared to those in other disciplines. (lit support) This is because the concepts and methods of statistical analysis are fundamental to the curriculum in many science and mathematics fields. Students in these disciplines are often exposed to statistical concepts and techniques early on in their studies and are required to use them regularly in their coursework and research. As a result, they may be more familiar and proficient with data analysis techniques

compared to students in other fields. Additionally, the nature of scientific and mathematical inquiry often requires the analysis and interpretation of data. Scientists and mathematicians are trained to be critical thinkers and approach problems logically and analytically. They are also taught to use statistical methods to draw conclusions and make predictions based on data. This emphasis on data analysis and interpretation may contribute to a more robust understanding of data literacy among science and maths students. Because of this, an important follow-up question arises: " “Are there discipline-related differences in perceptions on data literacy”

Table 4 shows differences in perceptions based on subject disciplines. STEM majors exhibit a noticeable enhancement in their perception of their abilities in understanding Basic Statistical concepts, basic Excel skills and data analysis, as compared to their non-STEM counterparts (see Table 4).

Table 4
Differences in perceptions towards understanding of statistical basic concepts based on disciplines

Survey	Components	Science and Math Mean (S.D.)	Other Disciplines Mean (S.D.)	Effect Size	p-value
Pre	Basic Statistical Concept	2.964(1.119)	2.585(1.298)	0.167	0.304
Post		3.626(1.040)	3.021(1.176)	0.286	0.076**
Pre	Basic Excel Skill	3.650(1.275)	3.222(1.366)	0.196	0.226
Post		4.021(0.978)	3.483(1.174)	0.325	0.043*
Pre	Data Analysis	2.806(1.273)	2.608(1.274)	0.032	0.855
Post		3.603(0.829)	2.972(1.174)	0.309	0.051**
Pre	Perception of Data Literacy	4.161(0.970)	4.111(1.204)	0.089	0.587
Post		3.958(1.091)	3.539(1.283)	0.238	0.141
Pre	Data Rationalization	3.896(1.011)	3.837(1.291)	0.106	0.515
Post		3.857(0.955)	3.546(1.240)	0.103	0.526

* statistically significant at 5% level; ** statistically significant at 10% level

Qualitative findings

In both surveys, pre- and post-boot camps, students were asked about their understanding of data literacy and whether it changed after the boot camps in the post-survey. In the pre-survey, they were also asked about what they want to learn in the boot camp, while in the post-survey they were asked about what they have enjoyed in the boot camp. Using a thematic analysis, we looked at students’ responses to open-ended questions in the surveys to identify, analyse, and interpret patterns of meaning or themes (Clarke & Braun, 2017) from the responses. We used an inductive approach in

generating the themes, where we are open with the flow and influence of the data (Fugard & Potts, 2020). It can be said that the process of generating themes is through "data-driven" analysis (Clarke & Braun, 2017).

Authors need to put details in this analysis, as in the qualitative findigs, it fail to show the process in the table 5

From the analysis of both surveys, students' understanding of data literacy are mainly developed around the notion of data literacy as the ability to work with data or information. Some emerging themes from students' responses are including the ability to read, analyse and understand the data, the ability to think based on data, the ability to learn and understand data, the ability to communicate data and the ability to analyse and generate conclusions from the data. The other strands of themes include a range of abilities related to data literacy such as the ability to collect or gather information as data, the ability to manage data, the ability to process meaningful information, and the ability to visualise data. Responding to the sub question in the post-survey about the change of their perception of data literacy after joining the boot camps, many students confirmed that they have better understanding about data literacy after joining the boot camp. They particularly highlighted increasing awareness about the importance of data literacy and practical insights from the intervention on how to work with data and to apply their conceptual understanding of data literacy.

percentage?

In the pre-survey, students were asked what they expected to learn in the boot camp. Many students showed their interest to learn data-related skills, such as how to analyse, present and manage the data, and practical skills of Microsoft Excel, such as using different formulas and functions. Nevertheless, few students still wanted to learn some basic statistical concepts, such as mean, median, mode and standard deviation. After the boot camp, in the post-survey, most students confirmed that the application of data-related skills and practical skills in using Microsoft Excel were parts of the boot camp they enjoyed learning the most. The practical insights such as how to collect, calculate, arrange, tidy up and process the data, as well as the use of pivot table, filter, functions and formulas in Microsoft Excel were materials in the boot camp that students found useful. Several students particularly mentioned that the data challenge was the most insightful part of the boot camps. These students emphasised that the project in the data challenge provided the opportunity to implement their knowledge of data literacy from using the concepts for generating meaningful information and making decisions. With a real case from the activities in the boot camps, they gained an important insight of how data literacy is useful for daily life.

How many, authors had 5 participants only

Details of themes and sub-themes generated from thematic analysis of the data from pre-boot camp survey are presented in Table 5 below.

Table 5
Themes and sub-themes generated from thematic analysis of the data from pre-boot camp surveys

Survey questions	Themes of responses
Describe your understanding the meaning of data literacy	• the ability to work with data or information<br>• the ability to read, analyse and understand data<br>• the ability to think based on data<br>• the ability to collect or gather information as data<br>• the ability to manage data<br>• the ability to process meaningful information<br>• the ability to communicate data
What would you like to learn in the boot camps?	• How to work with data<br>○ how to analyse the data<br>○ how to present the data<br>○ working with data more efficiently<br>○ creativity in working with data<br>○ classify the data<br>○ data management<br>• Using Microsoft Excel<br>○ Functions in excel<br>○ Formulas in excel<br>○ Learn spreadsheet<br>• Basic of statistics<br>○ Mean<br>○ Median<br>○ Mode<br>○ Standard deviation

Details of themes and sub-themes generated from thematic analysis of the data from post-boot camp survey are presented in Table 6 below.

Table 6
Themes and sub-themes generated from thematic analysis of the data from post-boot camp surveys

Survey questions	Themes of responses
Describe your understanding the meaning of data literacy	• an ability to work with data to generate meaningful information<br>• the ability to calculate data<br>• a set of individual ability and skill in reading, writing, communicating, counting and problem solving<br>• it is about managing data<br>• the ability to read, understand, create and communicate data<br>• the ability to understand the data<br>• the ability to visualize data<br>• the ability to read and analyse data<br>• It is about how we look at data<br>• an ability to obtain information from written data<br>• the ability to read, understand, create, and communicate data

	● it is about data manipulation and extract information from it
Has it changed after joining the boot camps	● Yes<br>● I know and understand better<br>● Yes, I understand more about data literacy<br>● Data literacy is very important<br>● Data literacy is really interesting and useful<br>● Data literacy is critical for entering workforce<br>● I become more aware of a lot of information<br>● I understand more about steps to process the data<br>● No, but I can work on data faster and more effectively<br>● No, but I can do data calculation very quickly
What have you enjoyed learning the most at the boot camps?	● Working with data<br>- how to arrange data<br>- how to process data<br>- tidy up data<br>- knowledge of data, collecting data, calculating data<br>- creating data<br>- comparing data<br>- techniques to speed up data processing<br>- when I do the filter for data<br>● Skills in using Microsoft Excel<br>- Pivot table<br>- Different functions in excel<br>- Using formulas in excel<br>- Filtering data in excel<br>- How excel works<br>● Data challenge<br>- work on data challenge<br>- the project<br>- the implementation of data literacy<br>- problem solving<br>● Basic concepts of statistics<br>- types of data<br>- knowledge about data

Students who agreed to join a focused group discussion had the opportunity to elaborate their opinion about the boot camp and their perspective about data literacy in general. When they were asked about the general impression of the boot camp, all students agreed that it was useful as it provided them with applied skills related to some basic statistical concepts. A highlight is particularly attributed to practical insights they gained from the intervention, which they found complementary to what they have learned at school. A student said, “We have learned basic statistics, such as mean and median, but from the boot camps, we just knew the application to make decisions based on the data.” Another student added, “For me, as at school, Excel was just introduced, so we just learned the basic skills, and joining the boot camps was very useful, so we knew the application. We just learned a few formulas at school, which were not discussed deeply”.

Discussion

Need to have more references from similar studies to support the argumentation in this section

The main objective of this paper is to investigate high school students' perceptions of their understanding of basic statistical concepts and data literacy skills after joining data literacy boot camp as an intervention. It also analyses the impact of the intervention and the perceived benefits of the skills learnt from the boot camp. The analysis of quantitative data shows that the student's perception of their understanding basic statistical concepts and data analysis skills have significantly improved after the intervention, while basic Excel skills have improved slightly. As shown in figure 1 above, most students perceived their understanding of basic statistical concepts was improved after joining the boot camp. As confirmed by participants of the focus group discussion, the use of real data from a real life problem and context help them to grasp the concepts more deeply. These findings confirm recommendations of the GAISE College Report (AMA, 2016) that suggest the integration of real data in a particular context for a particular purpose in teaching introductory statistics. This integration, in turns, can help learners to "develop the ability to think statistically" (AMA, 2016, p. 8)

In general, participants confirmed that their experience joining the boot camp helped them to raise their awareness about the importance of data literacy, which indicated that the objective of the intervention was achieved. Many responses in the post-survey confirmed that their perception about data literacy has changed after the boot camp, with emphasis on increasing awareness of the importance of data literacy in daily life. They became more aware of the availability and abundance of data in their surroundings (Suryadi et al., 2021). In the FGD one student confirmed, "The boot camp was very good, for me I can understand the use of data literacy for everyday life". This awareness, according to Suryadi et al. (2021), is something that generally is lacking among science students.

When students were asked in the FGD about their experience of joining the boot camp, one of the students said "We have learned basic statistics, such as mean and median at school, but from the boot camps, we just knew the application to make decisions based on the data". Another student added, "For me, as at school, Excel was just introduced, so we just learned the basic skills, and joining the boot camps was very useful, so we knew the application. We just learned a few formulas at school, which

were not discussed deeply”. These findings show that the design of the boot camps with materials and delivery that focused on understanding of basic concepts and their application is relevant for high school students, as suggested in the GAISE II (NCTM, 2020).

The use of real data sets in the boot camp also helps students to understand the relevance of the concepts in their daily life. As one student in the FGD stated, “The boot camp was very good … for me I can understand the use of data literacy for everyday life”. This is relevant to recommendations from the GAISE College Report (2016) that the use of real data sets will help students to have better conceptual understanding. This strategy was particularly prominent in the data challenge that is provided to participants at the end of the boot camp after all sessions have been delivered. Students reflected on the experience working on the data challenge as challenging yet exciting and insightful. They confirm that the experience allowed them to practise decision making that was informed by data.

Improvement in using different functions and formulas of Microsoft Excel is another practical skill that students find insightful and useful from the boot camps. Although the quantitative analysis showed only a slight improvement of students' perceived ability in using Microsoft Excel, the qualitative data indicated that students found the practice of using the tool very useful and this was the part they enjoyed the most in the boot camp. One of the FGD participants stated that “I just knew how powerful it (pivot table) was, it can process vast amounts of data very quickly”. As outlined in the GAISE report (AMA, 2016), the use of technology to analyse data is a main recommendation of how to teach introductory statistics courses in higher education.

The use of real data sets in the boot camp also helps students to understand the relevance of the concepts in their daily life. As one student in the FGD stated, “The boot camp was very good … for me I can understand the use of data literacy for everyday life”. This is relevant to recommendations from the GAISE College Report (2016) that the use of real data sets will help students to have better conceptual understanding.

Lack of references to support the argumentation, and also authors need to justify the methods for boot camp, since not every students could work well using zoom

Conclusions

Data literacy boot camps as an intervention to raise awareness of the criticality of data literacy skills are beneficial for students in secondary education. The findings of this study show that students perceive the intervention complements the understanding of some basic statistical concepts they have learned at school, as the boot camps improve their skills in applying the concepts to a real case and data set. Students got the opportunity to exercise their consciousness to assess the validity of data and how to use data to draw meaningful conclusions.

With the perceived impact and benefits confirmed by students above, it is recommended that the data literacy boot camps can be a strategic intervention to prepare secondary students for the transition to higher education and future career. Another pedagogical implication is that teachers can adopt learning materials and approaches in teaching statistical concepts. For policy-making, the study has implications for better advocacy to encourage schools to integrate data literacy into their curriculum.

Since this study was a pilot project, there is room for improvement in this project, both in the design and the scalability of the intervention. For the design, this pilot project can be contextualised in terms of its content and delivery method to match students' needs and conditions. For the scalability, there is a great opportunity to broaden the scope and coverage of the project to reach more secondary students in different contexts, in different regions in Indonesia and even adopted for contexts in different countries.

Acknowledgements

References

Carlson, J., Fosmire, M., Miller, C. C., & Nelson, M. S. (2011). Determining Data Information Literacy Needs: A Study of Students and Research Faculty. *Libraries and the Academy, 11*(2), 629–657.

Clarke, V., & Braun, V. (2017) Thematic analysis. *The Journal of Positive Psychology*, *12*(3), 297-298, DOI: 10.1080/17439760.2016.1262613

Cowie, B., & Cooper, B. (2017). Exploring the challenge of developing student teacher data literacy. *Assessment in Education: Principles, Policy & Practice, 24*(2), 147–163. https://doi.org/10.1080/0969594x.2016.1225668

Erwin, R. (2015). Data Literacy: Real-world learning through problem-solving with data sets. *American Secondary Education 43*(2), 18-26. Spring 2015.

Farr, D. E., Zeh, H. J., & Abdelfattah, K. R. (2021). Virtual boot camps: An emerging solution to the undergraduate medical education–graduate medical education transition. *JAMA surgery, 156*(3), 282-283.

Fugard, A., and Potts, H. W. W (2020). Thematic analysis. In Atkinson, P., Delamont, S., Cernat, A., & Williams, R.A. (Eds.). S*age Research Methods Foundations SAGE Publications Ltd*. DOI: https://doi.org/10.4135/9781526421036858333

American Statistical Association (ASA). (2016). *Guidelines for Assessment and Instruction in Statistics Education (GAISE) College Report 2016*. Endorsed by ASA Revision Committee, retrieved from http://www.amstat.org/education/gaise.

Gebre, E. H. (2018). Young adults' understanding and use of data: Insights for fostering secondary school students' data literacy. *Canadian Journal of Science, Mathematics and Technology Education. 18*, 330–341 https://doi.org/10.1007/s42330-018-0034-z

Irish, T., Berkowitz, A., & Harris, C. (2019). Data Explorations: Secondary Students' Knowledge, Skills and Attitudes Toward Working with Data. Eurasia *Journal of Mathematics, Science and Technology Education, 15*(6), em1686. https://doi.org/10.29333/ejmste/103063

Kamberelis, G., Dimitriadis, G., & Welker, A. (2018). Focus Group Research and/in Figured Worlds. In Denzin, N.K., & Lincoln, Y.S. (Eds.). *The SAGE HAndbook of Qualitative Research 5th Edition*. Thousand Oaks, SAGE Publications, Inc.

Makarim, N. (2021). *The power of education to transform societies: Fireside chat with Dr Ilham Habibie.* Retrieved from https://www.youtube.com/watch?v=jALdzmQQnAo&t=3512s

Ministry of Education, Culture, Research and Technology Republic of Indonesia (MoECRT). (2021). *Merdeka Belajar. Episode 1* https://merdekabelajar.kemdikbud.go.id/en/episode_1/web

Schield, M. (1999). Statistical literacy: Thinking critically about statistics. *Of Significance, 1*(1), 15-20.

Shreiner, T. L. & Dykes. B. M. (2021). Visualizing the teaching of data visualizations in social studies: A study of teachers' data literacy practices, beliefs, and knowledge. *Theory & Research in Social Education, 49*(2), 262-306. DOI: 10.1080/00933104.2020.1850382.

Stockwell et al., (2015)

Suryadi, Mahardika, I. K., Supeno, & Sudarti. (2021). Data literacy of high school students on physics learning. *Journal of Physics Conference Series ,1839*(1), 012025. doi:10.1088/1742-6596/1839/1/012025

The National Council of Teachers of Mathematics (NCTM). (2020). *The Pre-K–12 Guidelines for Assessment and Instruction in Statistics Education II (GAISE II): A Framework for Statistics and Data Science Education.* Alexandria, VA: American Statistical Association.

The Organisation for Economic Co-operation and Development (OECD). (2019). *Core Foundations for 2030. OECD Future of Education and Skills 2030*. Retrieved from www.oecd.org/education/2030-project.

Rizal, R., Rusdiana, D., Setiawan, W., Siahaan, P., & Ridwan, I.M. (2021) Gender differences in digital literacy among prospective physics teachers. *Journal of Physics: Conference Series. 1806* (1), 012004, DOI 10.1088/1742-6596/1806/1/012004.
