# Supplementary material for: Exploring data literacy self-perception among Indonesian high school students
Source: PLoS One. 2025 Jan 3;20(1):e0312306. doi: 10.1371/journal.pone.0312306 (PMC11698427; doi:10.1371/journal.pone.0312306)
Supplement: Attachment — Submitted filename: Response to reviewers PONE-D-24-17279.docx [file pone.0312306.s003.docx]

Response to reviewers’ recommendations

| **Comments** | **Reviewer 1** | **Reviewer 2** | **Response** |
| --- | --- | --- | --- |
| Methodology - research design | p.5 line 116  What is the research design that authors used in this study? research and development? or mixed methods? |  | A methodology section has been added in the manuscript, explaining the study in general including the research design, methods of data collection and data analysis (Page 6 line 150).  Also, explanations related to recruitment of participants and data literacy boot camps provided as sub sections ‘participants’ (Page 7 line 175). |
| Data collection - instrument | Page 7 line 154  Further explanation needed for the instrument used in this study: how you develop the instruments, and did the authors used/adopted a conceptual approach  Further details of instruments used and whether authors developed the instruments (any theory used to develop) or adopted from similar studies |  | Further explanation of how instrument used in this study was developed and the concept and theoretical framework being adopted has been provided in the sub section ‘data collection’ under methodology section (Page 7 line 262) |
| Focus Group Discussion - participants recruitment | Page 7 line 154  explain more on this, how you maintain the personal contact? was it FGD? or what? what the main issues explored? |  | Explanation about Focus Group Discussion is provided in the methodology section (Page 7 line 167) |
| Focus Group Discussion - justification of group formation |  | How the authors justify putting male and female students in one group of FGD? | Further information about Focus Group Discussion is provided in the methodology section (Page 7 line 167) |
| Language | Page 10 line 231  unclear, and some confusion with the This refers to? |  | The sentence has been rephrased |
| Language | Page 10 line 233  I am not sure with this claim, authors need to support the argument with more references |  | The sentence has been rephrased with more reference |
| Qualitative analysis | Page 12 line 278  Authors need to put details in this analysis, as in the qualitative findings, it fails to show the process in the table 5 |  | More details of qualitative analysis have been added (Page 17 line 415) |
| Data | Page 12 line 288  percentage |  | The data percentage has been provided |
| Data | Page 12 line 294  How many, authors had 5 participants only  Page 12 line 294  Several students |  | The statement has been revised |
| Bootcamp | Page 16 line 396  Lack of references to support the argumentation, and also authors need to justify the methods for boot camp, since not every students could work well using zoom | Describe more clearly how the bootcamp was delivered. It stated in the manuscript that it was 2 virtual sessions, @ 2 hours. However, it isn't clear yet who were the speakers, how specific topics were distributed and delivered in the first and second session? was it delivered during a school year period or in a school break? considering the school timeline/schedule which usually have determined by the school. | A subsection of data literacy boot camps has been added under the methodology section, explaining details of the bootcamps and addressing the reviewer’s comments. (Page 10 line 220) |
| Findings/Results | Qualitative analysis needs further details especially in presenting the qualitative results. It should match with data analysis. |  | More details of qualitative data have been added |
| Discussion | Argumentation in discussion section need to be improved by citation to similar or relevant studies. |  | More details of qualitative data have been added |
| Discussion | Starts Page 15 line 337  Need to have more references from similar studies to support the argumentation in this section |  | Further discussions with added literature have been added to the manuscript |
